# The role of perinatalcare in early life trauma prevention

**DOI:** 10.1192/j.eurpsy.2021.204

**Published:** 2021-08-13

**Authors:** T. Kurimay, J. Szederkenyi, A. Hortobagyi

**Affiliations:** Buda Family Centre Mh Centre Psychiatry, North Centre Buda New Saint John Hospital and Oupatient Clinic, Budapest, Hungary

## Abstract

**Abstract Body:**

Mental health support for parents, infants and children as an interdisciplinary, cross-sectoral task has existed for decades in many European countries. A highlighted goal of integrated services (medical, social and educational) is to support competent parenting and the positive parent-child relationship, for the optimal development including mental health of infants and children. In clinical practice, the role of psychiatrists is often linked to healing and rehabilitation, even though we also have an important role in prevention. The mental support, treatment and prevention of psychiatric disorders during the pre-, peri- and postnatal period are often not considered being preventive measures. The perinatal period is the most sensitive and at the same time one of the most important stages of our lives. The traumas suffered during this period affect both the mother and the newborn, in fact it affects the family as a whole. Models for the prevention of early trauma appear at the level of social community and, inter alia, health and social care. Traumas are closely linked to social determinants of health. Gene environment interactions also allow for the transgenerational transmission of trauma. The presentation introduces individual and family levels of interdisciplinary care, good practices and programs the knowledge of which may be important to psychiatrists. How the practicing psychiatrist can contribute to trauma prevention and how to understand the development of resilience. The presenter will detail good practices, and highlight the possibilities for all clinicians on ways to work in their respective field with a trauma preventive approach.

**Disclosure:**

No significant relationships.

